# Entropy Measures as Geometrical Tools in the Study of Cosmology

**DOI:** 10.3390/e20010006

**Published:** 2017-12-25

**Authors:** Gilbert Weinstein, Yosef Strauss, Sergey Bondarenko, Asher Yahalom, Meir Lewkowicz, Lawrence Paul Horwitz, Jacob Levitan

**Affiliations:** 1 Physics Department, Ariel University, Ariel 40700, Israel; 2 Department of Mathematics, Ariel University, Ariel 40700, Israel; 3 Department of Mathematics, Ben Gurion University, Be’er Sheva 84105, Israel; 4 Department of Electrical and Electronic Engineering, Ariel University, Ariel 40700, Israel; 5 School of Physics and Astronomy, Raymond and Beverly Sackler Faculty of Exact Sciences, Tel Aviv University, Tel Aviv 69978, Israel; 6 Department of Physics, Bar Ilan University, Ramat Gan 52900, Israel

**Keywords:** general relativity, cosmology, Raychaudhuri equations, entropy

## Abstract

Classical chaos is often characterized as exponential divergence of nearby trajectories. In many interesting cases these trajectories can be identified with geodesic curves. We define here the entropy by S=lnχ(x) with χ(x) being the distance between two nearby geodesics. We derive an equation for the entropy, which by transformation to a Riccati-type equation becomes similar to the Jacobi equation. We further show that the geodesic equation for a null geodesic in a double-warped spacetime leads to the same entropy equation. By applying a Robertson–Walker metric for a flat three-dimensional Euclidean space expanding as a function of time, we again reach the entropy equation stressing the connection between the chosen entropy measure and time. We finally turn to the Raychaudhuri equation for expansion, which also is a Riccati equation similar to the transformed entropy equation. Those Riccati-type equations have solutions of the same form as the Jacobi equation. The Raychaudhuri equation can be transformed to a harmonic oscillator equation, and it has been shown that the geodesic deviation equation of Jacobi is essentially equivalent to that of a harmonic oscillator. The Raychaudhuri equations are strong geometrical tools in the study of general relativity and cosmology. We suggest a refined entropy measure applicable in cosmology and defined by the average deviation of the geodesics in a congruence.

## 1. Introduction

Classical chaos is generally defined as exponential divergence of nearby trajectories causing instability of the orbits with respect to initial conditions or quite simply as high sensitivity to initial conditions. The extent of divergence is quantified in terms of Lyapunov exponents measuring the mean rate of exponential separation of neighboring trajectories.

The norm (for the Euclidean case, for example):

$$d\left(\tau \right)=\sqrt{\sum_{i=1}^{n}\delta x_{i}^{2}\left(\tau \right)}$$

is a measure of the divergence of two neighboring trajectories, where $\delta x_{i}$ is the *i*-th component of the displacement between two nearby trajectories at time *t*. The mean rate of exponential divergence is given by [1]:

$$\omega =\lim_{\begin{array}{c} \tau \to \infty \\ d(0)\to 0 \end{array}}\frac{1}{\tau }\ln\left(\frac{d(\tau )}{d(0)}\right).$$

The Kolmogorov entropy is related to the Lyapunov exponents. It gives a measure of the amount of information lost or gained by the system as it evolves [1]. It can be computed from the Lyapunov exponent by:

$$h_{K}=\int_{P}\sum_{\omega_{i}>0}\omega_{i}\;d\mu ,$$

which is the sum of all positive Lyapunov exponents averaged over some region of the phase space *P* with measure dμ.

One would naturally be interested in defining a measure for stochasticity in regions with divergence. The function d(τ) initially has an irregular behavior and evolves into a form in which the limit as τ→∞ of:

$$\frac{1}{\tau }\left(\frac{d(\tau )}{d(0)}\right)$$

converges to a value that depends on the initial conditions. Casartelli et al. [2] argued that this quantity is deeply related to the Kolmogorov entropy and also exhibits strong sensitivity to the initial conditions.

Benettin et al. defined a similar entropy [3] and calculated a Kolmogorov-like entropy for the Henon–Heiles system. However, we shall take a different route in this study.

There are many interesting physically-relevant examples for which the trajectories can be put into correspondence with geodesic curves, for example in problems in general relativity and in the conformal map of Hamiltonian potential models [4], with geodesic deviation described in terms of a Jacobi equation related to the curvature. In the following, we provide a relation between the Jacobi equation, the entropy (as defined above) and the geodesic equation itself.

Let there be given two nearby geodesics, *m* and *n*, and let τ be the affine parameter on the geodesics. For a point x with parameter τ on the geodesic *m*, one may define the geodesic deviation as the length of the shortest path from *m* to *n*. Let us denote this geodesic deviation by χ(τ). The Jacobi equation states that [5]:

(1) $$\frac{d^{2}\chi \left(\tau \right)}{d\tau^{2}}=-K\left(x\left(\tau \right)\right)\chi \left(\tau \right)$$

where *K* is the Gaussian curvature, and this simple form for the curvature is restricted to two-dimensional systems.

We employ the entropy defined by [6]:

(2) S=lnχ(τ)

From Equations (1) and (2), we derive:

(3) $$\ddot{S}\left(\tau \right)+{\left(\dot{S}\left(\tau \right)\right)}^{2}+K\left(x\left(\tau \right)\right)=0,$$

where $\dot{}=d/d\tau$. One may transform Equation (3) to a Riccati-type equation by letting $\dot{S}\left(\tau \right)=X\left(\tau \right)$:

(4) $$\dot{X}\left(\tau \right)+X{\left(\tau \right)}^{2}+K\left(x\left(\tau \right)\right)=0.$$

The general Riccati equation has the form:

(5) $$\dot{X}\left(\tau \right)=q_{0}\left(\tau \right)+q_{1}\left(\tau \right)X+q_{2}\left(\tau \right)X^{2}.$$

The solution to Equation (5) is $X=-\dot{u}/q_{2}u$ with *u* being the solution to the equation:

(6) $$\ddot{u}-T\dot{u}+Ru=0$$

In Equation (6), $R=q_{2}q_{0}$ and $T=q_{1}+{\dot{q}}_{2}/q_{2}$. We therefore obtain the equation:

(7) $$\ddot{u}+Ku=0,$$

This equation is the Jacobi equation in two dimensions.

For a flat space K=0, and Equation (3) takes the form:

(8) $$\ddot{S}\left(\tau \right)+\left(S\dot{(}\tau \right){)}^{2}=0,$$

This equation has some resemblance to the geodesic equation:

(9) $$\frac{d^{2}x^{\mu }}{d\tau^{2}}+\Gamma_{\sigma \rho }^{\mu }\frac{dx^{\sigma }}{d\tau }\frac{dx^{\rho }}{d\tau }=0$$

in particular if $\Gamma_{\rho \sigma }^{\mu }$ vanishes for ρ not equal to σ.

## 2. Application to Gravitation

Consider, in particular, the geodesic equation for a null geodesic in a double-warped spacetime:

$$ds^{2}=-\phi^{2}dt^{2}+a^{2}g_{ij}dx^{i}dx^{j}$$

where ϕ=ϕ(x), a=a(t), and *g* is independent of *t*. Consider further a variation of the geodesic with $\delta x^{i}=0$:

$$\delta s=\int \left(-\phi^{2}\dot{t}\delta \dot{t}+aa^{\prime }g_{ij}{\dot{x}}^{i}{\dot{x}}^{j}\delta t\right)d\tau =0$$

where ${}^{\prime }=d/dt$. Integrating by parts, one gets $\int (\phi^{2}\ddot{t}+aa^{\prime }g_{ij}{\dot{x}}^{i}{\dot{x}}^{j})\delta t\;d\tau =0$, which implies:

(10) $$\phi^{2}\ddot{t}+aa^{\prime }g_{ij}{\dot{x}}^{i}{\dot{x}}^{j}=0,$$

For a null geodesic, one has:

$$\phi^{2}{\dot{t}}^{2}=a^{2}g_{ij}{\dot{x}}^{i}{\dot{x}}^{j}.$$

Substituting into the geodesic Equation (10) above leads to:

(11) $$\ddot{t}+\frac{a^{\prime }}{a}{\dot{t}}^{2}=0.$$

We have achieved the equation, which formally is the same as the entropy Equation (8).

Taking into account that the universe is evolving in time, we study the entropy S(τ)=lnχ(τ) in a four-dimensional cosmological spacetime with a time-dependent metric. It is in fact a special case of a Robertson–Walker metric for a universe for which the space for a fixed time is a flat three-dimensional Euclidean space expanding as a function of time [7] (The model used here provides a simple illustration of the similarity between the geodesic equation and the entropy Equation (8), which is the main intent of this study. The work in [7] also splits the geodesic equation into a time part and a space part, but uses a different technique with the purpose of obtaining the cosmological redshift). The metric of the model is given by:

(12) $$ds^{2}=-dt^{2}+a{\left(t\right)}^{2}\left(dx^{2}+dy^{2}+dz^{2}\right).$$

The Christoffel symbols for the time components μ=0 are given by [7]:

$$\Gamma_{00}^{0}=\Gamma_{i0}^{0}=\Gamma_{0i}^{0}=0,\;\Gamma_{ij}^{0}=a\left(t\right)\dot{a}\left(t\right)\delta_{ij}.$$

By inserting these into the geodesic Equation (9), we obtain:

(13) $$\frac{d^{2}x^{0}}{d\tau^{2}}+a\left(t\right)\dot{a}\left(t\right)\delta_{ij}\frac{dx^{i}}{d\tau }\frac{dx^{j}}{d\tau }=0.$$

The Christoffel symbols for the spatial components (μ≠0) are:

$$\Gamma_{jk}^{i}=\Gamma_{00}^{i}=0,\;\Gamma_{j0}^{i}=\Gamma_{0j}^{i}=\frac{\dot{a}\left(t\right)}{a(t)}\delta_{j}^{i}.$$

and the spatial part of the geodesic equation takes the form:

(14) $$\frac{d^{2}x^{i}}{d\tau^{2}}+\frac{\dot{a}\left(t\right)}{a(t)}\delta_{j}^{i}\frac{dx^{i}}{d\tau }\frac{dx^{j}}{d\tau }=0.$$

Equations (13) and (14) constitute the splitting of the geodesic equation into the timelike and spacelike parts [7].

For particles moving freely under purely gravitational forces, one can find a freely falling coordinate system with the motion being a straight line in spacetime:

(15) $$\frac{d^{2}x^{a}}{d\tau^{2}}=0.$$

Here, τ is the proper time:

(16) $$d\tau^{2}=\eta_{\alpha \beta }dx^{\alpha }dx^{\beta }$$

For massless particles, the Right Hand Side (RHS) of Equation (16) vanishes [7], and we may use $\sigma =x^{0}$ as the parameter instead of τ. Photons follow null-geodesics, and we restrict ourselves to paths along the x-axis, i.e., $x^{\mu }\left(\sigma \right)=\left\{t\left(\sigma \right),x\left(\sigma \right),0,0\right\}$. With the metric given by (12) and $ds^{2}=0$, we obtain:

(17) $$-dt^{2}+a{\left(t\right)}^{2}dx^{2}=0.$$

This leads to the equation:

(18) $$\frac{dx}{d\sigma }=\frac{1}{a(t)}\frac{dt}{d\sigma }$$

By solving for dt/dσ and inserting the null-condition (17) into the time component for the geodesic equation [6], we finally achieve the equation:

(19) $$\frac{d^{2}t}{d\sigma^{2}}+\frac{\dot{a}\left(t\right)}{a(t)}{\left(\frac{dt}{d\sigma }\right)}^{2}=0.$$

This equation is formally identical to the entropy Equation (8).

It is noteworthy that a resemblance between the geodesic equation and the entropy equation is obtained by inserting the null condition into the time part of the geodesic equation and not the spatial part, which underlines the connection between the present definition of entropy with time rather than space.

## 3. The Raychaudhuri Equation

The definition of entropy as defined by Equation (2) has its origin in the geodesic deviation equation describing the behavior of a one-parameter family of nearby geodesics and is, as remarked, in the present form restricted to systems of at most two dimensions. For higher dimensional systems, one needs more refined tools to describe the behavior of a bundle of geodesics, the so-called congruence. We now argue that the Raychaudhuri equation may provide such tools in dimension four. In a forthcoming study, we shall show examples of entropy defined by the average deviation of the geodesics in a congruence.

Let $\xi^{i}$ be the tangent vector field to a geodesic flow and $h^{ij}$ be the metric on the subspace perpendicular to ξ. The Raychaudhuri equation is:

(20) $$\frac{d\theta }{d\tau }=-\frac{1}{3}\theta^{2}-\sigma_{ij}\sigma^{ij}+\omega_{ij}\omega^{ij}-R_{ij}\xi^{i}\xi_{j},$$

where τ is the affine parameter along the geodesic and $R_{ab}$ is the Ricci tensor of the metric [8], $\theta =\nabla_{i}\xi_{j}h^{ij}$ is the expansion, $\sigma_{ij}=\nabla_{(i}\xi_{j)}-\frac{1}{3}\theta h_{ij}$ the shear and $\omega_{ij}=\nabla_{[i}\xi_{j]}$ the twist. Round brackets represent symmetrization and square brackets represent anti-symmetrization.

For completeness, and because it is very simple, we carry out the derivation of this equation explicitly. Denoting the covariant derivative $\nabla_{j}\xi_{i}$ by $\xi_{ij}$, the geodesic equation is:

$$\xi^{j}\xi_{ij}=0,$$

and because $\xi^{i}\xi_{i}=\mathrm{constant}$, it follows that also:

$$\xi^{i}\xi_{ij}=0.$$

Without loss of generality, we assume that $\xi^{i}\xi_{i}=-1$. The metric on the spacelike subspace perpendicular to ξ is then:

$$h_{ij}=g_{ij}+\xi_{i}\xi_{j}.$$

We now decompose the derivative $\xi_{ij}$ of ξ into three components:

$$\begin{array}{cc} \theta & =h^{ij}\xi_{ij}=g^{ij}\xi_{ij},\\ \sigma_{ij} & =\frac{1}{2}\left(\xi ij+\xi ji\right)-\frac{1}{3}\theta h_{ij},\\ \omega_{ij} & =\frac{1}{2}\left(\xi_{ij}-\xi_{ji}\right). \end{array}$$

We note that the expansion θ measures the logarithmic derivative of the volume element in the space perpendicular to ξ; the shear $\sigma_{ij}$ measures the non-conformal part of the deformation of the metric *h*; and the twist $\omega_{ij}$ measures the entangling of the geodesic trajectories, i.e., the obstruction to ξ being hypersurface-orthogonal. The expansion θ in (20) corresponds to $\dot{S}$ in (2) and can be taken as the derivative of the entropy. Equation (2) is the two-dimensional version of (20).

We can decompose:

$$\xi_{ij}=\frac{1}{3}\theta h_{ij}+\sigma_{ij}+\omega_{ij},$$

and note that these three components are mutually orthogonal:

$$\frac{1}{3}\theta h_{ij}\sigma^{ij}=\frac{1}{3}\theta h_{ij}\omega^{ij}=\sigma_{ij}\omega^{ij}=0.$$

The first expression on the left vanishes because σ is traceless; the second and the third vanish because $h_{ij}$ and $\sigma_{ij}$ are symmetric, while $\omega_{ij}$ is anti-symmetric.

Taking a derivative of θ along ξ, we find:

$$\dot{\theta }=\nabla_{\xi }\left(g^{ij}\xi_{ij}\right)=\xi^{k}g^{ij}\xi_{ijk},$$

where for simplicity, we have denoted $\nabla_{k}\xi_{ij}=\xi_{ijk}$. From the definition of the Riemannian tensor, we have:

$$\xi_{ijk}-\xi_{ikj}=-R_{jkim}\xi^{m},$$

hence we get:

$$\dot{\theta }=g^{ij}\xi^{k}\xi_{ikj}-R_{km}\xi^{k}\xi^{m},$$

where $R_{km}=g^{ji}R_{jkim}$ are the components of the Ricci tensor. Furthermore:

$$g^{ij}\xi^{k}\xi_{ikj}=g^{ij}\nabla_{j}\left(\xi^{k}\xi_{ik}\right)-g^{ij}{\xi^{k}}_{j}\xi_{ik}=-\xi^{ki}\xi_{ik}.$$

Substituting the decomposition of ξ and using the orthogonality relations, we obtain:

$$-\xi^{ki}\xi_{ik}=-\frac{1}{9}\theta^{2}h^{ki}h_{ik}-\sigma^{ki}\sigma_{ik}+\omega_{ik}\omega_{ik}=-\frac{1}{3}\theta^{2}-\sigma^{2}+\omega^{2}.$$

Substituting back into the equation for $\dot{\theta }$, we obtain (20).

Consider now the Einstein equations $R_{\mu \nu }-\frac{1}{2}Rg_{\mu \nu }=8\pi GT_{\mu \nu }$. Taking the trace, we get R=−8πGT, hence substituting back into the Einstein equations, we obtain $R_{\mu \nu }=8\pi G\left(T_{\mu \nu }-\frac{1}{2}Tg_{\mu \nu }\right)$, and therefore, $R_{\mu \nu }U^{\mu }U^{\nu }=8\pi G\left(T_{\mu \nu }-\frac{1}{2}Tg_{\mu \nu }\right)U^{\mu }U^{\nu }$. Most known physical matter fields satisfy the Strong Energy Condition (SEC), which states that for all time-like vectors *U*, the inequality $T_{\mu \nu }U^{\mu }U^{\nu }\geq \frac{1}{2}Tg_{\mu \nu }U^{\mu }U^{\nu }$ holds. It follows, when the SEC holds, that the term $R_{\mu \nu }U^{\mu }U^{\nu }$ is always positive. Furthermore, note that the shear and the rotation are spatial vectors, and consequently, $\sigma_{\mu \nu }\sigma^{\mu \nu }\geq 0$ and $\omega_{\mu \nu }\omega^{\mu \nu }\geq 0$. As mentioned above, $\omega_{\mu \nu }$ is zero if and only if the congruence is hypersurface-orthogonal. If that is satisfied, the Raychaudhuri equation simplifies to the form:

(21) $$\frac{d\theta }{d\tau }+\frac{1}{3}\theta^{2}+\sigma^{2}=-R_{\mu \nu }U^{\mu }U^{\nu }.$$

In order for the Left Hand Side (LHS) to be negative, it must fulfill the condition $d\theta /d\tau <-\frac{1}{3}\theta^{2}$, which finally leads to the inequality:

(22) $$\frac{1}{\theta (\tau )}\geq \frac{1}{\theta_{0}}+\frac{1}{3}\tau$$

One concludes that any initially converging hypersurface-orthogonal congruence must continue to converge and within the finite proper time $\tau \leq -3\theta_{0}^{-1}$ will lead to crossing of geodesics (a caustic), which means that matter obeying the SEC cannot cause geodesic deviation, but will increase the rate of convergence in accordance with the fact that the SEC causes gravitation to be attractive [7]. The aim to define the entropy by the average convergence/divergence of the geodesics in a congruence will be tantamount to establish that the SEC will cause initially decreasing entropy to continue to decrease.

The Raychaudhuri equation for the expansion is a first-order nonlinear Riccati equation and hence of the same type as Equation (4) for which the solution, Equation (7), has the same form as the Jacobi equation.

If we set $\theta =3F^{\prime }/F$, the Raychaudhuri equation is transformed to:

(23) $$\frac{d^{2}F}{d\tau^{2}}+\frac{1}{3}\left(R_{\mu \nu }U^{\mu }U^{\nu }+\sigma^{2}-\omega^{2}\right)F=0,$$

which is a harmonic oscillator equation. As pointed out above, θ may be identified with the derivative of the entropy, so that according to (2) for the entropy S=lnF here, *F* may be identified with an effective geodesic deviation.

We recently proved [9] that the geodesic deviation equation of Jacobi is essentially equivalent to that of a harmonic oscillator. The expansion θ is the rate of growth of the cross-sectional area orthogonal to the bundle of geodesics. The increase/decrease of this area is the same as the divergence/convergence of the geodesics. The average growth of the cross-sectional area is compatible with the average geodesic deviation.

Kar and Sengupta have shown [8] that the condition for geodesic convergence is the existence of zeroes in Fat finite values of the affine parameter, and they argue that convergence occurs if:

(24) $$R_{\mu \nu }U^{\mu }U^{\nu }+\sigma^{2}-\omega^{2}\geq 0.$$

i.e., the shear accelerates convergence, and the rotation obstructs convergence.

## 4. Comments and Conclusions

Since shear transforms circles to ellipses, we compared the mean distance between uniformly-distributed pairs of independent points inside a circle to that inside an ellipse of the same area and found that it is smaller in the circle. The mean $\bar{d}$ of the distance *d* between pairs of points in a planar region Ω of area π can be computed by:

(25) $$\bar{d}=\frac{1}{\pi^{2}}\int_{\Omega }\int_{\Omega }\left|x-y\right|\;dA_{x}dA_{y}.$$

**Figure 1:**
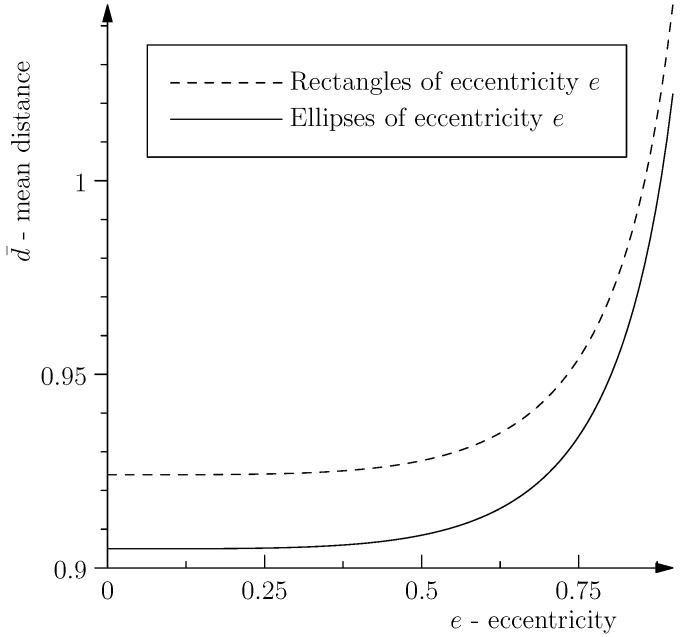
Mean distance between pairs of points.

The result is graphed against the eccentricity *e* in Figure 1. For comparison, we also computed the same quantity for rectangles of “eccentricity” *e* and area π, where by similarity with the definition for an ellipse, we defined the eccentricity of a rectangle with sides a≥b as $e=\sqrt{1-b^{2}/a^{2}}$. In fact, the mean distance between pairs of points inside any plane domain of area π is smallest for a circle, i.e., the circle is the unique minimizer of (25) among all planar regions of area π [10]. This might have important implications.

The evolution from an infinitesimal circular cross-section orthogonal to the flow lines to an elliptical one of the same area is brought about by shear. Moving the cross-section along the flow does not change the number of geodesics. However, due to the increase in the mean-distance between the geodesics when transforming from a circular to an elliptical cross-section, there is a diverging tendency of the geodesics moving along the flow. That implies, according to our proposed definition of entropy as the mean distance between geodesics in a bundle, that the evolution in the presence of shear exhibits an increase of entropy.

## References

[1] Tabor M. (1989). Chaos and Integrability in Nonlinear Dynamics: An Introduction.

[2] Casartelli M., Diana E., Galgani L., Scotti A. (1976). Numerical computations on a stochastic parameter related to the Kolmogorov entropy. Phys. Rev. A.

[3] Benettin G., Galgani L., Strelcyn J.M. (1976). Kolmogorov entropy and numerical experiments. Phys. Rev. A.

[4] Horwitz L., Zion Y., Lewkowicz M., Schiffer M., Levitan J. (2007). Geometry of Hamiltonian Chaos. Phys. Rev. Lett..

[5] Gutzwiller M. (1990). Chaos in Classical and Quantum Mechanics.

[6] Evangelidis E.A., Neethling J.D. (1983). On the existence of an entropy-like quantity. Astrophys. Space Sci..

[7] Carroll S. (2004). Spacetime and Geometry: An Introduction to General Relativity.

[8] Kar S., Sengupta S. (2007). The Raychaudhuri equations: A brief review. Pramana.

[9] Strauss Y., Horwitz L.P., Levitan J., Yahalom A. (2015). Quantum Field Theory of Classically Unstable Hamiltonian Dynamics. J. Math. Phys..

[10] Groemer H. (1982). On the average size of polytopes in a convex set. Geometr. Dedic..

